# Correspondences Between Music and Involuntary Human Micromotion During Standstill

**DOI:** 10.3389/fpsyg.2018.01382

**Published:** 2018-08-07

**Authors:** Victor E. Gonzalez-Sanchez, Agata Zelechowska, Alexander Refsum Jensenius

**Affiliations:** RITMO Centre for Interdisciplinary Studies in Rhythm, Time and Motion, Department of Musicology, University of Oslo, Oslo, Norway

**Keywords:** music-induced motion, sensorimotor synchronization, embodied cognition, movement analysis, motion capture, music information retrieval

## Abstract

The relationships between human body motion and music have been the focus of several studies characterizing the correspondence between voluntary motion and various sound features. The study of involuntary movement to music, however, is still scarce. Insight into crucial aspects of music cognition, as well as characterization of the vestibular and sensorimotor systems could be largely improved through a description of the underlying links between music and involuntary movement. This study presents an analysis aimed at quantifying involuntary body motion of a small magnitude (micromotion) during standstill, as well as assessing the correspondences between such micromotion and different sound features of the musical stimuli: pulse clarity, amplitude, and spectral centroid. A total of 71 participants were asked to stand as still as possible for 6 min while being presented with alternating silence and music stimuli: Electronic Dance Music (EDM), Classical Indian music, and Norwegian fiddle music (Telespringar). The motion of each participant's head was captured with a marker-based, infrared optical system. Differences in instantaneous position data were computed for each participant and the resulting time series were analyzed through cross-correlation to evaluate the delay between motion and musical features. The mean quantity of motion (QoM) was found to be highest across participants during the EDM condition. This musical genre is based on a clear pulse and rhythmic pattern, and it was also shown that pulse clarity was the metric that had the most significant effect in induced vertical motion across conditions. Correspondences were also found between motion and both brightness and loudness, providing some evidence of anticipation and reaction to the music. Overall, the proposed analysis techniques provide quantitative data and metrics on the correspondences between micromotion and music, with the EDM stimulus producing the clearest music-induced motion patterns. The analysis and results from this study are compatible with embodied music cognition and sensorimotor synchronization theories, and provide further evidence of the movement inducing effects of groove-related music features and human response to sound stimuli. Further work with larger data sets, and a wider range of stimuli, is necessary to produce conclusive findings on the subject.

## 1. Introduction

The intricate relationships between music and human body motion has been of interest to researchers for several decades, but recent technological developments have allowed for more robust and thorough studies, with works focusing on music-induced motion, music performance, and general sensorimotor synchronization (Gritten and King, 2006; Jensenius, 2007; Nusseck and Wanderley, 2009; Maes et al., 2014a; Su, 2016). Moreover, works on sensorimotor synchronization (SMS) have shown what appears to be a predisposition for humans to synchronize motion to periodic stimuli sequences even in the presence of continuous tempo changes (Repp and Su, 2013; van der Steen et al., 2015; Burger et al., 2017).

Many SMS studies have been based on tapping paradigms, but some have exploring other forms of moving in synchrony with external auditory rhythms, such as dance in humans (Keane, 2009; Solberg and Jensenius, 2017b) and synchronization to musical beat in vocal learning animals (Patel et al., 2009; Fitch, 2013). Janata et al. (2012) compiled a series of analysis methods to explore sensorimotor coupling and found that the feeling of being in “the groove” plays a fundamental role in musical appraisal. Furthermore, stimuli with a high level of groove elicit spontaneous rhythmic motion not only from the hands and fingers, but also other body parts such as the head and the legs (Madison, 2006; Kilchenmann and Senn, 2015).

Additionally, body motion related to SMS and groove has been found to follow structured patterns which are often in line with sound-producing actions (Küssner et al., 2014; Godøy et al., 2016).

The concept of embodied cognition assumes that cognitive processes require interactions between the body and its environment (Wilson, 2002). Studies of embodied music cognition propose the need of spontaneous body motion for musical meaning formation and the processing of musical features (Maes et al., 2014b), and a close relationship between spontaneous motion to music and predictions of pulse and rhythmic patterns. This approach to music and motion explains the reflecting and imitating qualities of motion to music as bidirectional processes, where body motion is not only a response to the music stimuli, but also part of the perception mechanism (Todd, 1999; Keller and Rieger, 2009; Witek et al., 2014). Sensorimotor synchronization, then, can be considered as one of the factors involved in this process, with Leman suggesting *embodied attuning* and *empathy* as the other two main components of embodied music cognition (Leman, 2008). It is through such embodied attuning that humans associate musical features such as melody, tonality, or timbre, with motion (Maes et al. 2014a). Empathy, on the other hand, allows for musical features to generate emotion and convey expressions (Wöllner 2012).

Building on the idea of body motion as a means for processing musical information, Phillips-Silver and Trainor (2008) have shown that when people move their body to a certain beat they are more able to interpret ambiguous rhythmic patterns. Moreover, they have demonstrated that body motion does not need to be voluntary to improve music cognitive processes. Participants were rocked on every second and third beat of an ambiguous auditory rhythm pattern while lying passively on a seesaw, and were afterwards asked to interpret the meter of the rhythmic stimuli. A second set of experiments compared passive motion of the head to passive motion of the lower limbs and found that only head motion improved the participants' rhythm encoding abilities. Based on these two studies the authors suggest that the effect of head motion on rhythm processing is due to the fundamental role of input from the vestibular system, and they further propose an underlying integration of auditory and vestibular inputs in the relationship between motion and auditory metrical rhythm perception.

The acoustic sensitivity of the vestibular system and its role in music cognition has been investigated in studies by Todd (1999), in which he observed that acoustic sequences with varying energy, amplitude, or pulse produce vestibular response signals, which, in turn, can produce a modulated sense of motion. Todd has also proposed a sensory-motor theory based on humans' experience of rhythm through both a *sensory representation* (of temporal information in the stimulus) and a *motor representation* (of own musculoskeletal system; motor image of the body). In this structure, the spatiotemporal characteristics of an acoustic stimuli are linked to the dynamic characteristics of the motor system, inducing an internal motion representation of the musculoskeletal system, even with actual motion not occurring. According to Todd's results and observations, the interplay between the vestibular and sensory-motor mechanisms is particularly evident when presented to stimuli with a highly variable range of acoustic features, such as in dance music.

Similarities between sound and motion in musical experience have been studied systematically by Godøy et al. (2016) by exploring the multimodal relations between sound and motion features. Music-related body motion has been generally categorized by the authors as either “sound-producing” or “sound-accompanying,” but with a wide overlap between the two categories. Moreover, the authors suggest that such music-related body motion can also be found in a scale between “quasi-stationary” postures and motion, with the postures serving as orientation points, commonly observed at downbeats and other accented points in the music. Studies by the same authors include a number of quantitative and qualitative analysis methods aimed at establishing correlations between physical sound and motion signals and the subjective perceptions of the related musical experiences. In one of such studies (Nymoen et al., 2013), the authors explore the relationships between sound and motion through a “sound-tracing” experiment in which the subjects moved their hands spontaneously to musical sound. Different sound “contours” (pitch, dynamics, timbre) were used for a correlation analysis with motion features of the participants' tracings.

Distinct time-varying sound and motion contour features were identified through Spearman correlation and canonical correlation analysis. The correlation coefficients allowed to measure the participants' temporal accuracy in mimicking the various sound features. The analysis methods proposed by the authors render additional evidence to the ample range of actions that people perform to sounds, and provide a baseline for the identification and classification of music-related motion.

Insight into human gestural descriptions of sound was also found in Caramiaux et al. (2014), with participants exposed to both causal and non-causal sounds, and asked to describe the stimuli through arm and hand gestures. Findings from this study rendered evidence of a fundamental effect of sound source identification in the subsequent gestural description. With causal sounds being generally described mimicking the perceived producing action. In this same line, in Küssner et al. (2014), differences in consistency in gestural representation of sound features were found between trained musicians and untrained participants in real-time exercises. This was particularly evident for pitch, being mostly represented with changes in height, and tempo, being described with changes in hand speed. These movement associations to sound provide initial evidence of consistent bodily responses to particular features, and raises questions over the effect of a wider range of sound characteristics and experimental conditions that have yet to be studied.

Following findings on the influence of rhythmic structures and periodicity on the amount of induced body motion, Burger et al. (2013) investigated relationships between musical features, such as rhythm, timbre, and tempo, with motion characteristics. Pulse clarity, percussiveness, and spectral flux were extracted from a series of stimuli and correlated with a number of free-motion features. Results from this study suggest that whole-body motion seem to be associated with a clear pulse in the music, while spectral flux and percussiveness seemed to have a larger influence on head and upper limb motion. No relationships were observed, however, between tempo and motion features. On the other hand in Styns et al. (2007) synchronization of walking with music was highest around 120 BPM tempo. The potential influence of tempo features on the amount of motion requires further investigation.

Closer to the topic of this paper, Ross et al. (2016) explored music and motion links of people standing still (what they call “quiet” standing) by recording fluctuations in the center of pressure (CoP) of 40 participants listening to music with low and high levels of groove. Events in CoP sway and in the music stimuli were cross-correlated to assess relationships between music and motion, while entrainment was analyzed using spectral coherence. The results suggest that the musical stimuli with a high level of groove produced the least amount of radial sway variability, and the musical experience was observed to influence the amount of postural variability and entrainment. Moreover, high groove was observed to favor entrainment of shorter rhythmic events. Such involuntary entrainment suggests an effect of involuntary musical entrainment on motor and balance control systems, and render additional evidence of involuntary and unconscious motion to music. The study provided additional evidence to factors contributing to the perception of groove, with changes in loudness, pulse clarity, and spectral flux being closely related to changes in perceptual groove. In the present study, an effort is made to further explore which of these—and other groove-related musical features—have the largest effect on motion remains to be fully assessed, as well as the relatively unexplored couplings between non-groove music stimuli and involuntary motion.

In Gandemer et al. (2014), the influence of rotating sound on standing balance was assessed through postural sway recordings from a force platform. Sway amplitude was found to be negatively correlated with the speed of the rotating sound. Subjects exhibited greater stability during fast rotating sound trials, compared to immobilized sound conditions. Although these findings were framed in the context of the role of the auditory system in postural regulation, insight from these results may also suggest the influence of the vestibular system in both sound processing and motion control. Moreover, Coste et al. (2018) found that discrete auditory rhythms have a significant effect in both voluntary and involuntary body sway, with entrainment of sway being higher for tempi at a frequency that was closer to the dominant sway frequency.

In a series of studies aimed at characterizing and understanding music-induced micromotion, Jensenius et al. (2017) investigated how music influences the motion of groups of participants trying to stand still. This micromotion is primarily involuntary and is performed at a scale that is barely observable to the human eye. The study consisted of a statistical comparison of measured motion between music and silent conditions and found that the subjects exhibited a remarkably consistent level of motion when attempting to stand still in silence (Jensenius, 2017). The measured standstill level of a person was shown to be consistent with repeated measures over time. The effects of different musical genres on standstill was measured by comparing Quantity of Motion (QoM) between 7 music excerpts, each with a duration of 20–40 s. The music stimuli were presented in ascending order of rhythmic complexity, starting with slow, non-rhythmical excerpts and ending with acoustic and electronic dance music. The study found significant differences in QoM between the music and silent conditions, with the largest mean QoM occurring during the EDM segment. Moreover, although horizontal motion (medio-lateral and anterior-posterior head sway) was found to account for most of the measured 3-dimensional QoM, vertical motion was shown to have clearer differences between music and silence conditions. These preliminary findings seem to provide additional evidence to findings by Burger et al. (2013) relating to the effects of spectral flux and percussiveness on head and upper limb motion.

In the following, we will describe an exploratory study designed to further characterize human music-related micromotion, with the aim of providing a quantification of the correspondences between music features associated with entrainment and micromotion, while at the same time aiding in the general understanding of sensorimotor theories as a natural manifestation of the internal motor engagement.

## 2. Materials and methods

### 2.1. Participants

A total of 71 participants took part in the study (33 female, 38 male, average age: 25 years, SD: 9.5 years) in groups consisting of 3–13 participants at a time^1^. The data collection took place during the University of Oslo “Open Day” in March 2017. The experiment was advertised as “The Norwegian Championship of Standstill” with a NOK 1000 prize for the participant with the lowest recorded motion. Recruitment was open to everyone with no exclusion criteria. Each participant was asked to report on the hours per week spent on the following activities: listening to music (16.8, SD: 12.2), creating music (4.7, SD: 5.3), dancing (1.1, SD: 1.3), and exercising (3.9, SD: 3.7). All participants gave their informed consent prior to the experiment and they were allowed to withdraw from the study at any point in time. The study obtained ethical approval from the Norwegian Center for Research Data (NSD), with the project identification number NSD2457.

### 2.2. Music stimuli

The participants were presented with segments of silence and music throughout the 6-min trials. All trials began and ended with 30 s of silence, followed by 5 min of alternating 60-s segments of music and silence. Thus, a complete sequence consisted of: Silence (30 s), Music1 (60 s), Silence (60 s), Music2 (60 s), Silence (60 s), Music3 (60 s), Silence (30 s). The three musical stimuli (played in random order for each group) were excerpts of:

1. Electronic Dance Music (EDM): the “break routine” of the track Icarus (Leclercq 2012). It is an example of a contemporary, energizing dance track with a clear pulse and even rhythmic pattern. This track has also been used in motion capture studies of dancers in Solberg and Jensenius (2017a,b).
2. Classical Indian music: a vocal improvisation by Tejaswinee Kelkar on top of a continuous drone from a shruti box. The track has a slow pulse, and a less clear rhythmic structure. This track has also been used in studies of sound-tracing in Kelkar and Jensenius (2017).
3. Norwegian folk music: a performance of traditional Telespringar dance music played on Hardanger fiddle. This is an example of a piece with an asymmetrical beat pattern, characterized by a long–medium–short duration patter. This track has also been used in studies of rhythmic reference structures (Haugen, 2017).

**Figure 1:**
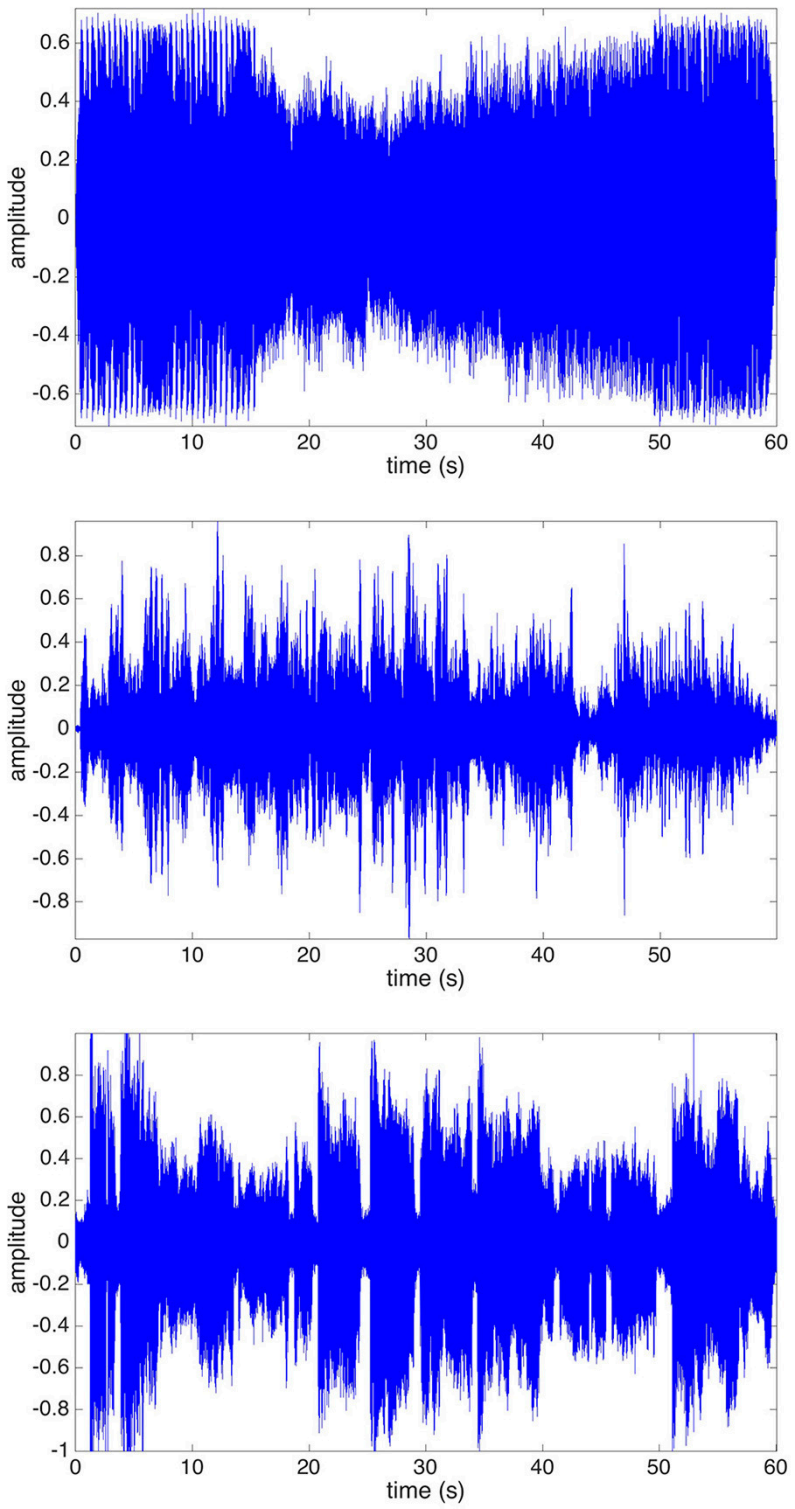
Waveforms of the presented music stimuli: **(Top)** Electronic Dance Music (EDM); **(Middle)** Norwegian fiddle music (Telespringar); **(Bottom)** Indian Classical Music.

The tracks were chosen so as to comprise different musical genres and features, and enable the exploration of global musical parameters. Figure 1 shows waveforms of the samples, to illustrate the dynamic differences of the tracks. The 60-s duration of the stimuli was chosen to allow participants enough time to engage with the music, while keeping the experiment sufficiently short to reduce the effect of tiredness. The sound was played comfortably loud from two Genelec 8020 loudspeakers and a Genelec 7050 sub-woofer.

### 2.3. Data acquisition

The instantaneous position of a reflective marker placed on each participant's head was recorded using a Qualisys infrared motion capture system (13 Oqus 300/500 cameras) running at 200 Hz. Previous studies have shown that the spatial noise level of this motion capture system is considerably lower than that of human standstill (Jensenius et al., 2012). Motion data was recorded and preprocessed in the Qualisys Track Manager (QTM), and the analysis was done in Matlab using the MoCap Toolbox (Burger and Toiviainen, 2013) and custom made scripts.

### 2.4. Procedure

**Figure 2:**
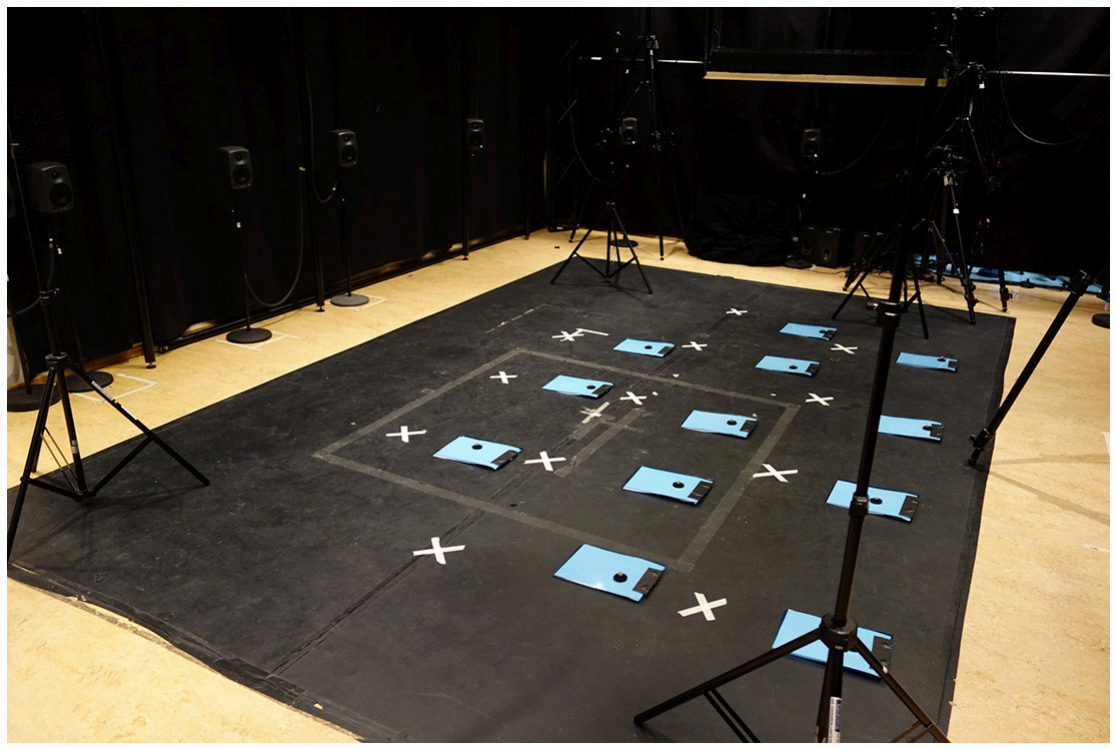
The setup for the experiment in the motion capture laboratory. White marks and questionnaires indicate the position of each participant. Poles with reference markers where placed in each corner of the capture space and used to check the noise-level of the recording (see Jensenius et al., 2012 for a description of the noise-level in optical motion capture systems).

The participants were recorded in groups of 3–13 people at a time. They were asked to stand as still as possible for 6 minutes, being free to choose their own standing position. The distribution of the participants in the laboratory was standardized across trials, with marks on the floor indicating the approximate feet position (Figure 2).

### 2.5. Quantity of motion

In order to measure the general standstill level, the *quantity of motion* (QoM) of each head marker was computed as the sum of all the position differences of consecutive samples of the marker, that is, the first derivative of the position time series:

$$\begin{array}{l} QoM=\frac{1}{T}\overset{N}{\underset{n\;=\;2}{\sum }}∥p\left(n\right)-p(n-1)∥ \end{array}$$

where *p* is either the two-dimensional (Z axis—the vertical plane) or three-dimensional (XYZ axes) position vector of a marker, *N* is the total number of samples and *T* is the total duration of the recording. The resulting QoM is measured in millimeters per second (mm/s). Instantaneous quantity of motion was obtained for each participant and for each stimulus.

### 2.6. Musical features

To investigate the correspondences between individual musical features and standstill micromotion, we performed computational feature extraction analysis of the presented music stimuli using the MATLAB MIRToolbox (version 1.6.2) (Lartillot et al., 2008a,b). Overall pulse clarity and tempo were obtained for each music stimuli, along with three time-varying frame-decomposed features that have been previously shown to contribute to motor entrainment to music and musical groove (Stupacher et al., 2014; Ross et al., 2016; Burger et al., 2017):

- Loudness: the dynamic envelope of the sound was obtained by calculating the RMS value of the frame-decomposed audio waveform (50 ms frame length).
- Brightness: measured as the spectral centroid of the frame-decomposed audio waveform, that is, the barycenter of the frequency spectrum (50 ms frame length).
- Pulse Clarity: calculated as the rhythmic clarity, indicating the strength of the beats (1 s frame length).

The similarity and correspondences between the sound and motion features were measured by computing cross-correlation between the moving averaged QoM time series (50 ms window length) for every participant and the extracted frame-decomposed musical features. The delay between the sound and motion feature signals was defined as the lag of maximum cross-correlation.

## 3. Results

### 3.1. Average quantity of motion

The average level of micromotion during the experiment, measured as the QoM of the entire set of participants, was QoM_mean_ = 8.76 mm/s. The standard deviation, QoM_SD_ = 2.20 mm/s, indicates a fairly low variability among participants. In fact, the extreme measurements across participants were QoM_max_ = 13.96 mm/s and QoM_min_ = 5.98 mm/s. These findings are in accordance with our previous findings on the general level of micromotion in human standstill (Jensenius et al., 2017).

When comparing the average QoM values to demographics, an independent-samples t-test indicated no significant differences between male and female participants [*t*_(69)_ = −1.69, *p* = 0.09]. Significant correlation was found between QoM and the participants' height, both during music (*r* = 0.34, *p* = 0.007) and during silent conditions (*r* = 0.32, *p* = 0.003), indicating that taller participants tended to move more during the whole experiment. Additionally, age had a significant negative correlation with QoM during the silent segment (*r* = −0.23, *p* = 0.034), while the correlation during the music segment was not significant (*r* = −0.17, *p* = 0.087).

The reported amount of hours per week spent doing physical exercise (group average = 3.9, SD = 3.7), creating music (group average = 4.6, SD = 5.3), and listening to music (group average = 17.2, SD = 12.6) had no significant correlation with measured QoM.

The participants were allowed to choose their standing posture during the experiment. In a post-experiment questionnaire they were asked to report on whether they were standing with their eyes open or closed, and whether they had their knees locked. The majority of the participants reported that they stood with open eyes (*N* = 58 vs. *N* = 1 for closed eyes, and *N* = 12 for those who switched between open and closed eyes during the experiment). Furthermore, 33 of the participants reported standing with locked knees, 23 switched between open and locked knees and 15 reported standing with unlocked knees. Two simple linear regression models were fit to predict QoM based on knee and eye strategy respectively. A significant regression equation was found for knee strategy, with *F*_(1, 68)_ = 3.7, *p* = 0.029, and an *R*^2^ of 0.072. Participants' QoM was approximately 1.31 mm/s smaller when standing with unlocked knees than when standing with locked knees. This also fits with previous findings (Jensenius, 2017). The regression equation predicting QoM based on eye strategy was found not significant [*F*_(1, 68)_ = 2.67, *p* = 0.076, *R*^2^ = 0.046].

### 3.2. Influence of music

The musical influence on the level of standstill was preliminarily assessed by computing the average QoM for the silence vs. music segments. The average for the music condition was QoM_mean_ = 8.83 mm/s (QoM_SD_ = 1.91 mm/s), while the average for the silent condition was QoM_mean_ = 8.57 mm/s (QoM_SD_ = 1.66 mm/s). A paired-samples (music and silence) t-test revealed that these differences were statistically significant [*t*_(70)_ = −2.89, *p* = 0.003].

**Figure 3:**
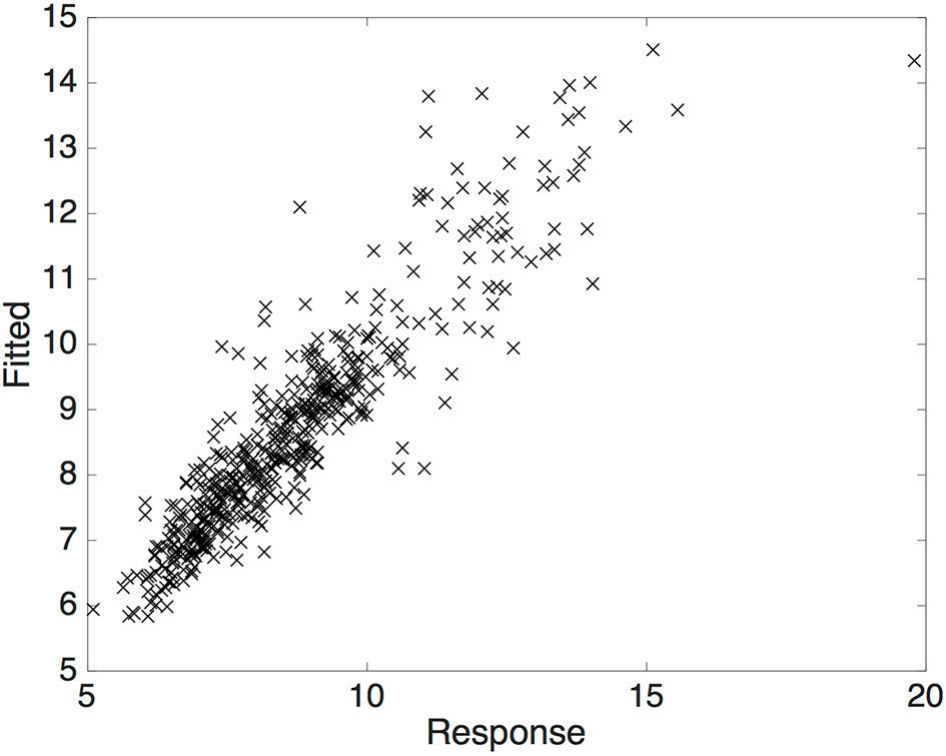
The fitted vs. observed response values from the linear mixed effects model form an almost 45-degree angle indicating a good fit.

A linear mixed effects model was fit to further analyze the effects of music on QoM. Stimuli was entered as fixed effects (first to last silent segments, EDM, Indian classical, and Telespringar) (Figure 3). The model was made of a random slope for by-subject effect of condition (Music or Silence) and a random intercept for Group. P-values were obtained by likelihood ratio tests between the full model (with the fixed effect) and a null model without the effect [χ^2^_(1)_ = 31.143, *p* < 0.001]. Bayesian information criterion (BIC) was used as penalized likelihood method for model selection, with smaller BIC number indicating better model adequacy (Table 1). Models tested included random slope for by-subject and by-group effect of Stimuli, as well as random intercepts for Subject, Condition, and Position in the capture volume. Random intercepts were also tested through standard deviation and confidence intervals with zero-crossings indicating that position in the lab and condition had no significant effect as random intercepts. The tests of fixed effects showed that EDM (*t* = 4.09, *p* < 0.001) has a significant effect on a participant's QoM (increasing it by approximately 0.65 mm/s, SE = ±0.16), but the remaining stimuli do not (*p* = 0.087).

**Table 1:** Bayesian information criterion (BIC) values for the penalized likelihood model selection.

**Model ID**	**Fixed effect**	**Random slope**	**Random intercept**	**BIC**
1	Stimuli	by-subject, by-group effect of stimuli	Subject, Group	1844
2	Stimuli	by-subject effect of stimuli	Subject, Group	1679
3	Stimuli	by-group effect of stimuli	Subject, Group	1748
4	Stimuli	by-subject, by-group effect of condition	Subject, Group	1645
5	Stimuli	by-subject effect of condition	Subject, Group	1609
6	Stimuli	by-group effect of condition	Subject, Group	1624
7	Stimuli	by-subject, by-group effect of stimuli	Group	1838
8	Stimuli	by-subject effect of stimuli	Group	1673
9	Stimuli	by-group effect of stimuli	Group	2199
10	Stimuli	by-subject, by-group effect of condition	Group	1639
**11**	**Stimuli**	**by-subject effect of condition**	**Group**	**1603**
12	Stimuli	by-group effect of condition	Group	2065
13	Stimuli	by-subject, by-group effect of stimuli	Subject	1838
14	Stimuli	by-subject effect of stimuli	Subject	1674
15	Stimuli	by-group effect of stimuli	Subject	1742
16	Stimuli	by-subject, by-group effect of condition	Subject	1639
17	Stimuli	by-subject effect of condition	Subject	1605
18	Stimuli	by-group effect of condition	Subject	1618

*Model 11 had the smallest BIC number indicating better model adequacy*.

### 3.3. Musical features

#### 3.3.1. Correspondences with 3-D motion

Overall, the effect of the musical stimuli on motion seems to correspond with the higher tempo (126 BPM) and total pulse clarity (0.63) of the EDM stimulus, as compared to the other two stimuli (Table 2).

**Table 2:** Average music and motion features for each of the presented stimuli.

**Stimuli**	**Tempo (bpm)**	**Pulse clarity**	**QoM mean (mm/s)**	**QoM SD (mm/s)**
Telespringar	108.63	0.08	8.63	1.82
Indian	53.79	0.04	8.68	2.01
EDM	125.99	0.63	9.19	2.27

To further investigate the effect of musical features on the induced motion, cross-correlation was performed between the three-dimensional QoM measurements and the three sound features described in section 2.6 (loudness, brightness and pulse clarity) for the whole set of participants.

**Figure 4:**
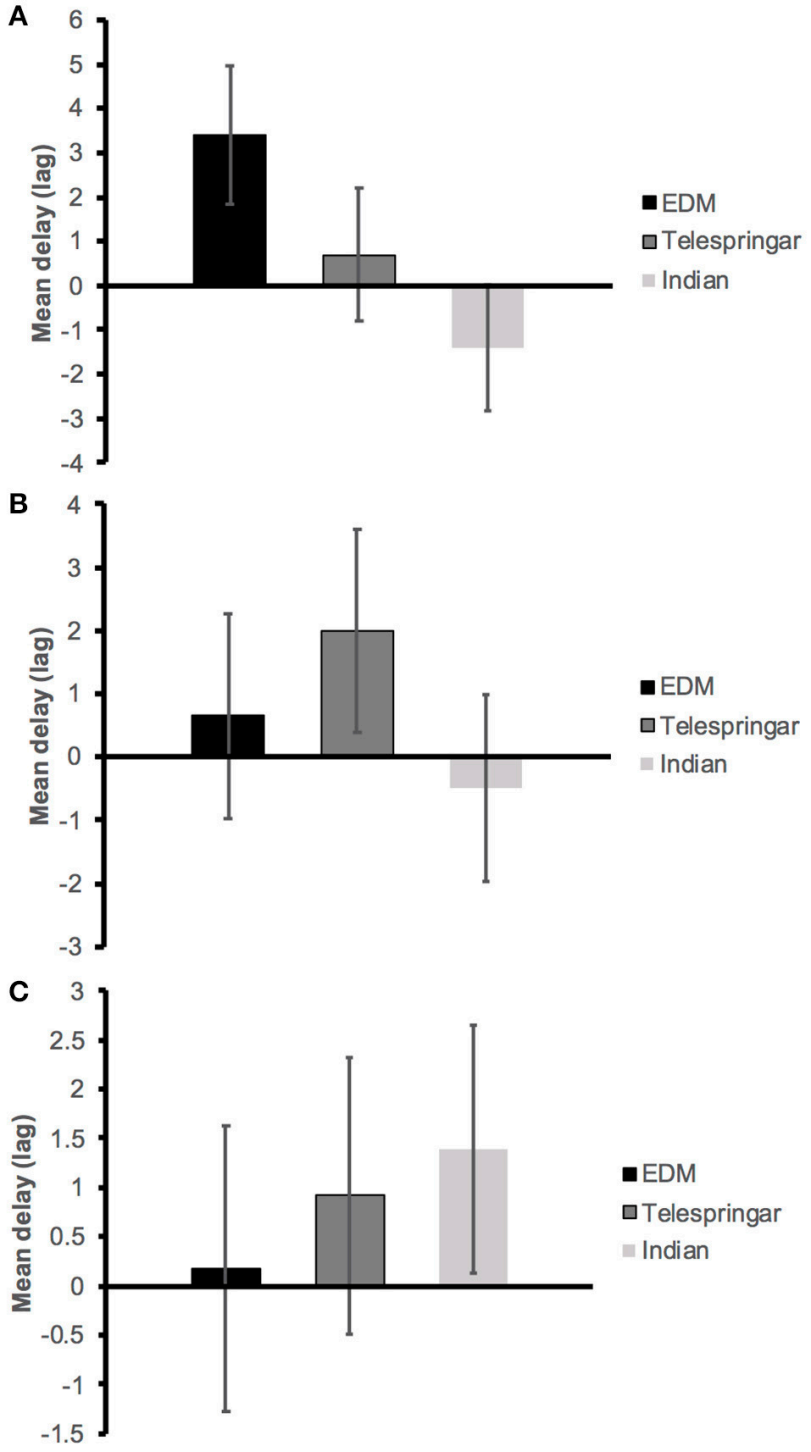
Average lag of maximum cross-correlation (delay) between *three-dimensional* QoM and extracted sound features across participants for each music stimuli. **(A)** Loudness (RMS), **(B)** Spectral centroid, **(C)** Pulse Clarity.

The EDM stimulus was observed to have the largest averaged lag of maximum cross-correlation (delay), at 3.39 ± 1.55 between loudness (measured as the RMS of the amplitude) and QoM. The Indian stimulus produced the smallest cross-correlation at lag −1.43 ± 1.41, indicating a degree of anticipation (Figure 4A). Interaction between conditions was assessed through one-way ANOVA with delay between QoM and RMS as dependent variable and music stimuli as independent variable. The effect of the stimuli on the correspondence between QoM and RMS approached significance at the 0.05 level [*F*_(2, 210)_ = 2.64, *p* = 0.07, η_p_^2^ = 0.025].

The Telespringar condition had the largest averaged lag of maximum cross-correlation between spectral centroid and QoM (2.01 ±1.60), while the Indian condition had the smallest (−0.49 ± 1.48), as shown in Figure 4B. The ANOVA showed no statistically significant differences between conditions when comparing correspondence between spectral centroid and QoM [*F*_(2, 210)_ = 0.63, *p* = 0.53, η_p_^2^ = 0.006].

Additionally, there were no statistically significant differences between conditions when comparing the frame-decomposed pulse clarity with QoM [*F*_(2, 210)_ = 0.20, *p* = 0.82, η_p_^2^ = 0.002]. The Indian music condition resulted in the largest averaged delay at 1.39 ± 1.27, while the EDM segment had the smallest delay at 0.17 ± 1.45 (Figure 4C).

#### 3.3.2. Correspondences with vertical motion

In order to investigate whether proposed connections between music features and vertical motion (Rusconi et al., 2006; Eitan et al., 2014) hold true also for the micromotion during standstill, as well as to further explore preliminary findings by Jensenius et al. (2017) on such correspondences, the vertical component of QoM was cross-correlated with frame-decomposed sound features.

**Figure 5:**
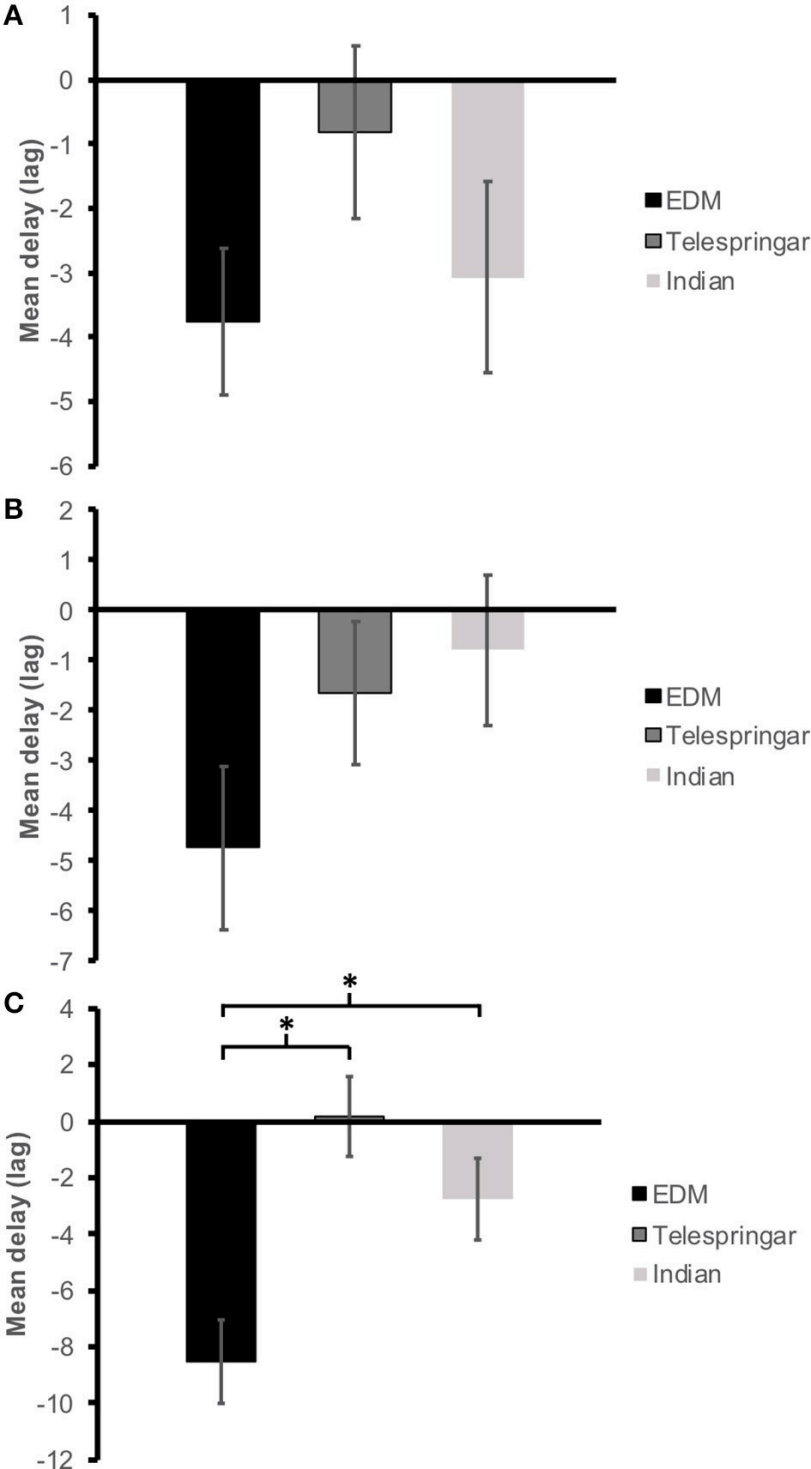
Average lag of maximum cross-correlation (delay) between *vertical* QoM and extracted sound features across participants for each music stimuli. **(A)** Loudness (RMS), **(B)** Spectral centroid, **(C)** Pulse Clarity. Asterisk indicates significant difference at *p* < 0.05.

The average lag of maximum cross-correlation between RMS and vertical QoM was maximum for the Telespringar music condition at −0.82 ± 1.34, while it was minimum for the EDM segment at −3.76 ± 1.14. The average delay was negative for the three conditions (Figure 5A), indicating a level of vertical motion anticipation to RMS events. No statistical significance was found on these differences across conditions at the 0.05 level [*F*_(2, 210)_ = 1.35, *p* = 0.26, η_p_^2^ = 0.013].

Correspondences between frame-decomposed spectral centroid and vertical QoM was maximum for the Indian music condition at −0.8 ± 1.51, while EDM had the largest anticipation at −4.73 ± 1.63, and all three conditions produced a negative average delay (Figure 5B). ANOVA revealed no statistical significance of the average delay differences between conditions [*F*_(2, 210)_ = 1.84, *p* = 0.16, η_p_^2^ = 0.017].

Finally, differences in delay between pulse clarity and vertical QoM were shown significant between conditions [*F*_(2, 210)_ = 9.27, *p* < 0.001, η_p_^2^ = 0.081], with the largest negative average delay occurring during the EDM segment at −8.52, while during the Telespringar stimulus the average delay was positive at 0.2 (Figure 5C). A Tukey post hoc test revealed that the delay between pulse clarity and vertical QoM was statistically significantly different during the EDM stimulus (−8.52 ± 1.50) when compared with the delay during both the Indian (−2.73 ± 1.46, *p* = 0.034) and the Telespringar (0.2 ± 1.42, *p* < 0.001) stimuli. There was no statistically significant differences between the delay during Telespringar and the delay during Indian music (*p* = 0.33).

## 4. Discussion

In this study we investigated the influence of music on human motion during standstill. Participants were presented with stimuli alternating between music excerpts and silence in order to determine the effect of music on their micromotion. The computed first derivative of head displacement (used here to represent quantity of motion, QoM) was significantly larger during the music condition, rendering additional evidence to findings from previous studies on music-induced micromotion by Jensenius et al. (2017), where participants were shown to move significantly more while exposed to music than during silent periods. Additionally, the current study expands on Jensenius et al. (2017) by further exploring the correspondences of entrainment-associated musical features with involuntary body motion in the 3-dimensional space and in the vertical plane.

The linear mixed effects model showed that the stimulus with a higher tempo and overall pulse clarity (EDM) produced more involuntary sway from head motion data. This is in line with findings by Ross et al. (2016), in which more motion entrainment to short rhythmical events was observed with increasing levels of groove in the stimuli. Moreover, results from the present study are also comparable to a certain degree with results by Burger et al. (2017), where clear pulses in the music stimuli were shown to correlate with free body movement features. Along with the aforementioned studies, findings from Janata et al. (2012), revealing spontaneous body reaction to high groove music, provide supporting evidence to the greater effect of EDM on involuntary body motion when compared to the other two stimuli.

The analysis of the correspondence between music and motion was performed by computing the delay between 3D and vertical QoM time-series and three frame-decomposed sound features strongly related to music groove perception: RMS, spectral centroid, and pulse clarity. The differences in average delay between the loudness envelope (RMS) and 3D QoM approached significance, while no significant differences were found for the vertical QoM data. Since all the tracks were perceptually normalized in sound level prior to the experiment, the loudness measurement may here be seen as an indication of the “denseness” of the musical material. The EDM condition had the largest delay, while the Indian music condition resulted in a negative delay which may be interpreted as anticipation. The lack of significant results for differences in correspondences between loudness and QoM, despite the range of stimuli that were presented, might suggest RMS has a low contribution to the overall feeling of entrainment.

No significant differences between stimuli were found when analyzing cross-correlation between spectral centroid (brightness) and both 3D and vertical QoM. Delay between spectral centroid and QoM was negative for vertical QoM across all stimuli. The observed negative delay pattern for vertical motion across stimuli could suggest a level of anticipation to perceived brightness events in the music. In line with these results, Nymoen et al. (2013) found negative correlation between vertical sound tracing gestures and spectral centroid, interpreted as a tendency of participants to represent changes in brightness with vertical motion. In the present study, involuntary anticipatory vertical motion to changes in sound brightness could be related to the participants' instantaneous perception of this feature, since there were no differences in motion across the diverse stimuli.

The statistically significant differences in delay between conditions for pulse clarity and vertical QoM can be interpreted as additional evidence to the effect of pulse clarity in music-induced motion. In particular, the anticipatory nature of the vertical motion, as evidenced by a relatively large negative delay during the EDM segment, corresponds with the overall greater pulse clarity of this stimulus when compared to the other two stimuli used in the study. Furthermore, the delay between vertical motion and pulse clarity events for the Telespringar music condition was the smallest, corresponding with the smallest overall pulse clarity of the stimulus. Although no significant delay differences were found between music conditions for 3D motion and pulse clarity, the average delay was positive across stimuli, as opposed to the mostly negative lag of maximum cross-correlation for vertical QoM. The different patterns between vertical and 3D motion across stimuli may be an indication of horizontal motion occurring as a response to pulse clarity events. Such a relationship between pulse clarity and involuntary motion might add to findings by Stupacher et al. (2014), where the wish to move the body to a musical pulse (defined as being “in the groove”) was strongly correlated with pulse clarity. Furthermore, in Ross et al. (2016), the involuntary sway of the center of pressure of participants was shown to entrain stronger to stimuli with a higher groove level, characterized by higher spectral flux, density, and pulse clarity.

The results from this study render additional insight into the underlying factors of embodied music cognition, particularly regarding involuntary correspondences between motion and different types of musical stimuli. The findings of correspondences between motion and the loudness envelope, brightness, and pulse clarity are partially in line with results from a number of studies on entrainment (Ross et al., 2016), sensorimotor synchronization (Janata et al., 2012), and the sensation of groove (Stupacher et al., 2014) and could complement such works with the inclusion of the quantification of correspondences to non-rhytmic and “non-groovy” stimuli. Follow-up studies will focus on further exploring the relationship between pulse clarity and vertical motion by testing smaller differences in pulse clarity across stimuli, as well as investigating correspondences with within-stimulus pulse clarity variability.

Capturing only the motion of a participant's head may be seen as a crude representation of a complex bodily interaction with music. The findings, however, proved consistent with our previous results (Jensenius et al., 2017), and also in line with the findings of Phillips-Silver and Trainor (2008), in which the role of the vestibular system in rhythm perception was observed through both passive and active head motion. Further studies on the correspondences between motion and a variety of sound features could contribute to a more robust characterization of the role of head motion for the perception and understanding of sound.

The music excerpts used in the present study were deliberately selected to cover diverse genres and different musical characteristics. Future studies should include other types of genres but also more examples within each genre. It will also be interesting to take into consideration the participants' musical preferences to better assess differences in musical taste, as the propensity to move might be dependent on liking of the stimulus. The extracted musical features used in the present study were selected based on other studies in the field of music-induced motion (Janata et al., 2012; Stupacher et al., 2014; Ross et al., 2016). They do not, however, represent the whole set of music characteristics that could prove relevant for sensorimotor synchronization and embodied cognition. Further work will aim at characterization of a larger set of features across stimuli.

Finally, while the present study focused on temporal characteristics of body motion, it may also be relevant to look at correspondences of music and motion frequencies, along with a wider range of physiological features such as heart rate, breathing patterns, skin conductance, and muscular activity, in order to further characterize involuntary bodily responses to music.

## Author contributions

VG-S, AZ, and AJ contributed conception and design of the study. VG-S, AZ, and AJ performed the experiments. VG-S pre-processed the data and performed the statistical analysis. VG-S wrote the first draft of the manuscript. VG-S, AZ, and AJ wrote sections of the manuscript. All authors contributed to manuscript revision, read and approved the submitted version.

### Conflict of interest statement

The authors declare that the research was conducted in the absence of any commercial or financial relationships that could be construed as a potential conflict of interest.

## Footnotes

^1^Due to the nature of the recruitment, group size was determined by demand and thus, varied as described. Familiarity between participants varied for each group. As described in the following sections, the Group factor was tested as one of the effects in the linear mixed effects model and the result was not significant.
